# *Vital Signs*: Trends in Emergency Department Visits for Suspected Opioid Overdoses — United States, July 2016–September 2017

**DOI:** 10.15585/mmwr.mm6709e1

**Published:** 2018-03-09

**Authors:** Alana M. Vivolo-Kantor, Puja Seth, R. Matthew Gladden, Christine L. Mattson, Grant T. Baldwin, Aaron Kite-Powell, Michael A. Coletta

**Affiliations:** ^1^Division of Unintentional Injury Prevention, National Center for Injury Prevention and Control, CDC; ^2^Division of Health Informatics and Surveillance, Center for Surveillance, Epidemiology, and Laboratory Services, CDC.

## Abstract

**Introduction:**

From 2015 to 2016, opioid overdose deaths increased 27.7%, indicating a worsening of the opioid overdose epidemic and highlighting the importance of rapid data collection, analysis, and dissemination.

**Methods:**

Emergency department (ED) syndromic and hospital billing data on opioid-involved overdoses during July 2016–September 2017 were examined. Temporal trends in opioid overdoses from 52 jurisdictions in 45 states were analyzed at the regional level and by demographic characteristics. To assess trends based on urban development, data from 16 states were analyzed by state and urbanization level.

**Results:**

From July 2016 through September 2017, a total of 142,557 ED visits (15.7 per 10,000 visits) from 52 jurisdictions in 45 states were suspected opioid-involved overdoses. This rate increased on average by 5.6% per quarter. Rates increased across demographic groups and all five U.S. regions, with largest increases in the Southwest, Midwest, and West (approximately 7%–11% per quarter). In 16 states, 119,198 ED visits (26.7 per 10,000 visits) were suspected opioid-involved overdoses. Ten states (Delaware, Illinois, Indiana, Maine, Missouri, Nevada, North Carolina, Ohio, Pennsylvania, and Wisconsin) experienced significant quarterly rate increases from third quarter 2016 to third quarter 2017, and in one state (Kentucky), rates decreased significantly. The highest rate increases occurred in large central metropolitan areas.

**Conclusions and Implications for Public Health Practice:**

With continued increases in opioid overdoses, availability of timely data are important to inform actions taken by EDs and public health practitioners. Increases in opioid overdoses varied by region and urbanization level, indicating a need for localized responses. Educating ED physicians and staff members about appropriate services for immediate care and treatment and implementing a post-overdose protocol that includes naloxone provision and linking persons into treatment could assist EDs with preventing overdose.

## Introduction

The opioid overdose epidemic continues to worsen in the United States. In 2016, a total of 63,632 drug overdose deaths occurred, a 21.4% increase from 2015 (*1*,*2*). Nearly two thirds (66.4%) of drug overdose deaths in 2016 involved prescription opioids, illicit opioids, or both, an increase of 27.7% from 2015 (*2*). Heroin and synthetic opioids (e.g., fentanyl) are driving increases in opioid-involved deaths (*2*–*4*). Tracking opioid overdoses is important to informing targeted interventions; however, timely national data on opioid overdoses evaluated in emergency departments (EDs) have been unavailable. Hospital billing data from 2014 indicate that approximately 92,000 ED visits occurred for unintentional, nonfatal opioid overdoses (*5*), but the time lag poses challenges to monitoring and response. ED syndromic data are important for tracking public health outbreaks (*6*) and can potentially identify changes in opioid overdoses quickly. Compared with billing data, syndromic data are collected in near real-time and can be viewed within 24–48 hours of an ED visit. ED syndromic data can serve as an early warning system to alert communities to a rise in opioid overdoses. Given the rapid availability of ED syndromic data, spikes in ED overdose trends are important to monitor and can potentially predict future fatal overdose trends and inform a more localized response. In addition, persons who experience an overdose are more likely to have a subsequent overdose (*7*); thus, EDs provide a crucial opportunity to link patients to treatment to avoid repeat overdoses. This report examines changes in opioid overdoses seen in the ED according to regional, state, and urbanization levels, to identify and track opioid overdoses and inform response efforts and recommendations for ED physicians and staff members.

## Methods

ED visits* from CDC’s National Syndromic Surveillance Program (NSSP)^†^ and Enhanced State Opioid Overdose Surveillance (ESOOS)^§^ program were analyzed to track trends in suspected unintentional or undetermined^¶^ opioid overdoses (opioid overdoses) by quarter and U.S. region (Northeast, Southeast, Southwest, West, and Midwest)** during July 2016–September 2017. NSSP receives demographic and chief complaint data and *International Classification of Diseases, Tenth Revision, Clinical Modification* (ICD-10-CM) diagnostic codes for approximately 60% of ED visits^††^ in the United States (*8*,*9*). Only visits involving patients aged ≥11 years were analyzed because they account for the majority of overdoses (*2*). NSSP ED data were analyzed using the Electronic Surveillance System for the Early Notification of Community-based Epidemics (ESSENCE) software. ED visits with ICD-10-CM diagnosis codes T40.0–T40.4, T40.6, T40.69, F11.12, F11.22, or F11.92; or chief complaint text indicating opioid use, “opioid,” and a word or abbreviation indicating an overdose (e.g., “OD”) were classified as suspected opioid overdoses.^§§^ To account for changes occurring across time and region, quarterly trends for the percentage of ED visits involving suspected opioid overdoses (ED visits involving opioid overdoses divided by total ED visits and multiplied by 10,000) were analyzed and stratified by sex, age group, and U.S. region. Quarterly rate changes were calculated for all quarters. Yearly change, controlling for seasonal effects, was estimated as the change from third quarter 2016 to third quarter 2017. Significance testing was conducted using chi-square tests. Average linear quarterly percentage change was calculated for each strata using a joinpoint regression program.^¶¶^

Whereas NSSP includes syndromic data from a large number of states, the lowest level of aggregation is at the regional level, without additional approval from each state.*** Hence, ESOOS syndromic and hospital billing data were analyzed at the state and county level to identify suspected opioid overdoses during July 2016–September 2017 in 16 funded states (Delaware, Illinois, Indiana, Kentucky, Maine, Massachusetts, Missouri, New Hampshire, New Mexico, Nevada, North Carolina, Ohio, Pennsylvania, Rhode Island, West Virginia, and Wisconsin), providing a more localized view. Three states used the NSSP suspected opioid overdose definition and 13 states developed their own definitions to capture the specific text and diagnoses used in their hospitals. Quarterly percentage change in rates are presented by state and county urbanization level^†††^ and analyzed as described.

## Results

**Figure 1:**
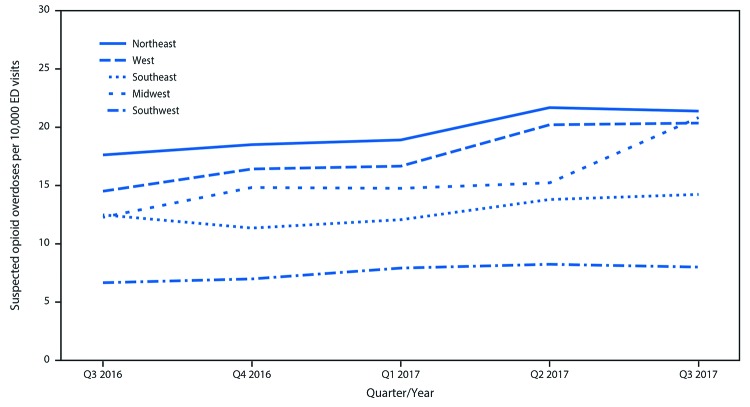
Quarterly rate* of suspected opioid overdose, by U.S. region^†^ — 52 jurisdictions in 45 states, National Syndromic Surveillance Program, July 2016–September 2017^§^ **Abbreviation:** ED = emergency department. * Per 10,000 ED visits. ^†^ *Northeast Region:* HHS Region 1 (Maine, Massachusetts, New Hampshire, Rhode Island, and Vermont), Region 2 (New Jersey and New York), and Region 3 (District of Columbia, Maryland, Pennsylvania, Virginia, and West Virginia); *Southeast Region:* HHS Region 4 (Alabama, Florida, Georgia, Kentucky, Mississippi, North Carolina, South Carolina, and Tennessee); *Southwest Region:* HHS Region 6 (Arkansas, Louisiana, New Mexico, and Texas); *Midwest Region:* HHS Region 5 (Indiana, Illinois, Michigan, Minnesota, Ohio, and Wisconsin) and Region 7 (Iowa, Kansas, Missouri, and Nebraska); *West Region:* HHS Region 8 (Colorado, Montana, North Dakota, and Utah), Region 9 (Arizona, California, and Nevada) and Region 10 (Alaska, Idaho, Oregon, and Washington). ^§^ Data current as of December 13, 2017.

Among approximately 91 million ED visits captured in NSSP during July 2016–September 2017, a total of 142,557 (15.7 per 10,000 visits) were suspected opioid overdoses. Opioid overdose ED visits in NSSP increased 29.7% from third quarter 2016 (July–September) to third quarter 2017; all five U.S. regions experienced prevalence increases (Figure 1), with the largest in the Midwest (69.7%), followed by the West (40.3%), Northeast (21.3%), Southwest (20.2%), and Southeast (14.0%) (Table 1). Substantial increases occurred among all demographic groups during the same period, including males (30.2%), females (24.0%), and persons aged 25–34 years (30.7%), 35–54 years (36.3%), and ≥55 years (31.9%). Most regions, age groups, and both sexes also experienced significant positive linear trends across all five quarters.

**TABLE 1:** Change in quarterly rates*^,†^ for suspected opioid overdose, by U.S. region,^§^ sex, and age group — 52 jurisdictions in 45 states, National Syndromic Surveillance Program, July 2016–September 2017^¶^

Characteristic	% Change					Average quarterly % change (95% CI)
	Q3 2016–Q4 2016	Q4 2016–Q1 2017	Q1 2017–Q2 2017	Q2 2017–Q3 2017	Q3 2016–Q3 2017	
**Overall**	**3.89**	**2.43**	**13.15**	**7.68**	29.65**	5.6 (1.8 to 9.5)**
**U.S. Region**						
Northeast	5.01	2.17	14.67	-1.40	21.30**	4.7 (-2.4 to 12.2)
Southeast	-9.08	6.32	14.29	3.21	14.03**	5.5 (0.6 to 10.6)**
Southwest	4.85	13.35	4.12	-2.87	20.19**	11.4 (1.1 to 22.9)**
Midwest	20.84	-0.48	3.19	36.73	69.67**	9.2 (4.1 to 14.6)**
West	13.11	1.50	21.28	0.75	40.28**	6.9 (3.4 to 10.5)**
**Sex**						
Male	6.21	2.62	10.66	7.96	30.21**	6.8 (4.4 to 9.2)**
Female	1.93	2.01	11.9	6.57	23.99**	5.8 (2.3 to 9.4)**
**Age group (yrs)**						
15–24	-1.11	-2.69	9.46	1.87	7.31**	2.1 (-1.6 to 5.9)
25–34	5.63	3.65	10.23	8.28	30.67**	6.9 (4.7 to 9.1)**
35–54	6.17	3.72	11.81	10.70	36.28**	8.0 (5.0 to 11.0)**
≥55	9.33	1.03	12.50	6.17	31.93**	7.1 (4.3 to 9.9)**

**Abbreviation:** CI = confidence interval.

* Per 10,000 emergency department visits.

^†^ Using the indicator counts and denominators, a rate of ED visits for each quarter was created using the count of suspected opioid overdose ED visits divided by the total number of ED visits for each quarter. Percentage change in rates subtracted the prior quarter from the current quarter then divided by the prior quarter multiplied by 100%.

^§^ The Northeast region includes HHS Regions 1 (Maine, Massachusetts, New Hampshire, Rhode Island and Vermont), 2 (New Jersey and New York), and 3 (District of Columbia, Maryland, Pennsylvania, Virginia, and West Virginia); the Southeast region includes HHS Region 4 (Alabama, Florida, Georgia, Kentucky, Mississippi, North Carolina, South Carolina, and Tennessee); the Southwest region includes HHS Region 6 (Arkansas, Louisiana, New Mexico, and Texas); the Midwest region includes HHS Regions 5 (Indiana, Illinois, Michigan, Minnesota, Ohio, and Wisconsin) and 7 (Iowa, Kansas, Missouri, and Nebraska); and the West region includes HHS Regions 8 (Colorado, Montana, North Dakota, and Utah), 9 (Arizona, California, and Nevada), and 10 (Alaska, Idaho, Oregon, and Washington).

^¶^ Data current as of December 13, 2017.

** Statistically significant (p*<*0.05).

Among approximately 45 million ED visits reported by the 16 ESOOS states from July 2016 through September 2017, a total of 119,198 (26.7 per 10,000 visits) were suspected opioid overdoses. Opioid overdose ED visits increased 34.5% from third quarter 2016 to third quarter 2017 (Table 2). Ten states experienced significant increases in prevalence during this period, although substantial variation was observed among states in the same region. For example, in the Northeast, significant increases occurred in Delaware (105.0%), Pennsylvania (80.6%), and Maine (34.0%), but other states, including Massachusetts, New Hampshire, and Rhode Island experienced nonsignificant (<10%) decreases. In the Southeast, a significant increase (31.1%) occurred in North Carolina, a significant decrease (15.0%) occurred in Kentucky, and a small, nonsignificant decrease (5.3%) was observed in West Virginia. In the West, a significant increase (17.9%) occurred in Nevada. All states in the Midwest reported significant increases, including Wisconsin (108.6%), Illinois (65.5%), Indiana (35.1%), Ohio (27.7%), and Missouri (21.4%).

**TABLE 2:** Change in quarterly and annual rates*^,†^ for suspected opioid overdose, by state — 16 states,^§^ Enhanced State Opioid Overdose Surveillance program, July 2016–September 2017^¶^

Region/State	% Change					Average quarterly % change (95% CI)
	Q3 2016–Q4 2016	Q4 2016–Q1 2017	Q1 2017–Q2 2017	Q2 2017–Q3 2017	Q3 2016–Q3 2017	
**Overall**	**8.91**	**9.09**	**13.06**	**0.12**	34.49**	8.4 (4.8 to 12.0)**
**Northeast**						
Delaware	8.77	10.95	43.00	18.76	104.95**	20.9 (10.5 to 32.2)**
Maine	2.57	-8.13	29.45	9.81	33.95**	7.9 (-2.4 to 19.3)
Massachusetts	-8.48	-11.48	3.11	18.97	-0.62	-1.0 (-11.4 to 10.6)
New Hampshire	-4.33	-17.91	29.67	-8.76	-7.09	-0.8 (-12 to 11.7)
Pennsylvania	29.79	17.51	25.89	-5.94	80.59**	17.0 (5.6 to 29.7)**
Rhode Island	2.80	4.54	5.44	-11.91	-0.18	0.9 (-5.0 to 7.2)
**Southeast**						
Kentucky	-26.94	40.45	3.52	-20.02	-15.04**	0.5 (-16.3 to 20.6)
North Carolina	-0.43	3.28	15.20	10.63	31.05**	7.4 (1.8 to 13.4)**
West Virginia	43.31	-16.64	4.02	-23.77	-5.28	-2.5 (-19.3 to 17.9)
**Southwest**						
New Mexico	26.11	1.51	-5.01	-10.93	8.30	1.2 (-10.4 to 14.4)
**Midwest**						
Illinois	23.13	1.48	2.82	28.80	65.47**	11.1 (2.7 to 20.1)**
Indiana	-10.15	11.20	10.45	22.43	35.11**	8.4 (-1.9 to 19.8)
Missouri	4.77	-1.77	9.54	7.67	21.38**	4.7 (1.2 to 8.3)**
Ohio	22.74	25.67	21.67	-31.94	27.74**	9.6 (-12.2 to 36.7)
Wisconsin	17.12	67.28	3.22	3.14	108.58**	22.3 (4.2 to 43.7)**
**West**						
Nevada	13.69	-9.46	11.37	2.82	17.88**	3.4 (-2.3 to 9.5)

**Abbreviation:** CI = confidence interval.

* Per 10,000 emergency department visits.

^†^ Using the indicator counts and denominators, a rate of ED visits for each quarter was created using the count of suspected opioid overdose ED visits divided by the total number of ED visits for each quarter. Percentage change in rates subtracted the prior quarter from the current quarter then divided by the prior quarter multiplied by 100%.

^§^ Delaware, Illinois, Indiana, Kentucky, Maine, Massachusetts, Missouri, Nevada, New Hampshire, New Mexico, North Carolina, Ohio, Pennsylvania, Rhode Island, West Virginia, and Wisconsin.

^¶^ Data current as of January 8, 2018.

** Statistically significant (p<0.05).

**Figure 2:**
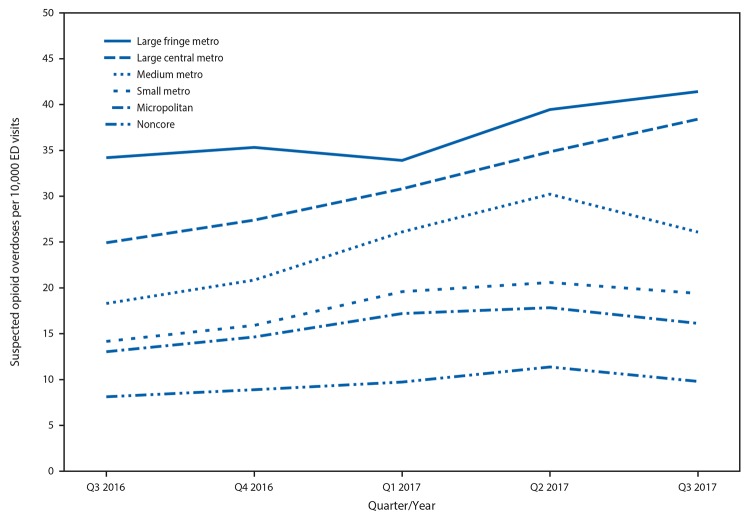
Quarterly rate* of suspected opioid overdose, by level of county urbanization**^†^****^,^****^§^** — 16 states,**^¶^** Enhanced State Opioid Overdose Surveillance program, July 2016–September 2017** **Abbreviation:** ED = emergency department. * Per 10,000 ED visits. ^†^ The six classification levels for counties were 1) large central metro: part of a metropolitan statistical area with ≥1 million population and covers a principal city; 2) large fringe metro: part of a metropolitan statistical area with ≥1 million population but does not cover a principal city; 3) medium metro: part of a metropolitan statistical area with ≥250,000 but <1 million population; 4) small metro: part of a metropolitan statistical area with <250,000 population; 5) micropolitan (nonmetro): part of a micropolitan statistical area (has an urban cluster of ≥10,000 but <50,000 population); and 6) noncore (nonmetro): not part of a metropolitan or micropolitan statistical area. ^§^ The average linear quarterly percentage change (QPC) was significant for large central metro (average QPC = 11.7, 95% confidence interval [CI] = 10.7 to 12.7, p<.001). QPCs for large fringe metro (average QPC = 5.1, 95% CI = −0.3 to 10.7); medium metro (average QPC = 11.4, 95% CI = −1.3 to 25.8); small metro (average QPC = 9.3, 95% CI = −0.1 to 19.5); micropolitan (average QPC = 6.4, 95% CI = −3.1 to 16.9); and noncore (average QPC = 6.4, 95% CI = −2.8 to 16.5) were not significant. ^¶^ Delaware, Illinois, Indiana, Kentucky, Maine, Massachusetts, Missouri, New Hampshire, New Mexico, Nevada, North Carolina, Ohio, Pennsylvania, Rhode Island, West Virginia, and Wisconsin. ** Data current as of January 8, 2018.

All urbanization levels experienced large and significant increases in ED opioid overdose visits from third quarter 2016 to third quarter 2017, including large central metropolitan (54.1%), medium metropolitan (42.6%), small metropolitan (36.9%), micropolitan (23.6%), large fringe metropolitan (21.1%), and noncore (20.6%) areas. Large central metropolitan areas experienced significant linear increases (Figure 2).

## Discussion

Despite data from the 2016 National Survey on Drug Use and Health indicating that heroin use and opioid misuse might be stabilizing (*10*), this analysis suggests that prevalence of suspected opioid overdose ED visits substantially increased in NSSP (29.7%) and ESOOS (34.5%) states from third quarter 2016 to third quarter 2017. Increases in ESOOS states were greater than those in NSSP states, which is likely driven by the higher mortality burden of drug overdose in ESOOS states (*2*). The increases occurred in most demographic groups and U.S. regions and suggest a worsening of the epidemic into late 2017 in several states, possibly related to the wide variation in the availability and potency of illicit drug products (e.g., fentanyl sold as or mixed into heroin) that increase overdose risk and drive increases in mortality (*3*,*4*,*11*). Enhanced prevention and treatment efforts in the ED and access to evidence-based opioid use disorder treatment, including medication-assisted treatment and harm reduction services, are needed (*12*).

The sharp increases and variation across localities indicate that real-time data are needed to better detect and respond to overdose spikes and to facilitate response coordination for regional or multiple state outbreaks. Enhanced data sharing among contiguous localities is needed because regional variation in drug products often cross state or county borders (*11*). Increases in the Midwest in NSSP and all five Midwestern ESOOS states (Illinois, Indiana, Missouri, Ohio, and Wisconsin) are consistent with opioid overdose death trends (*2*). However, increases in prevalence of ED visits for suspected opioid overdoses in the Southwest and West and decreases in the Southeast (Kentucky and West Virginia) were unanticipated and might foreshadow changes in opioid overdose death trends in 2017. The significant decreases in Kentucky might be explained by fluctuations in drug supply and warrant confirmation. In the Northeast, several states reported small decreases (Massachusetts, New Hampshire, and Rhode Island) or large increases (Delaware, Maine, and Pennsylvania) that are consistent with early 2017 drug overdose death reports from these states,^§§§^ possibly related to implementation of interventions including expansion of access to medication-assisted treatment.^¶¶¶^

The increases in opioid overdose rates in ESOOS metropolitan counties, specifically in large central (54.1%), medium (42.6%), and small metropolitan (36.9%) counties from third quarter 2016 to third quarter 2017 are consistent with previous reports indicating that heroin overdose hospitalizations, ED visits, and deaths were highest in metropolitan areas (*2*–*5*). Two of the three areas with highest rates of heroin overdose deaths, large central metropolitan and medium metropolitan areas (*2*), reported the sharpest increases in opioid overdose ED visits, highlighting the need for targeted efforts to reduce the burden of opioid overdose in these areas and slow or reverse increases in overdoses driven by changes in the illicit opioid drug market. The magnitude of opioid pain reliever misuse and heroin use, however, only varies slightly across urbanization levels, and all urbanization levels report increases in ED visits for opioid overdoses (*5*). Thus, generalized public health interventions tailored to each community context are necessary.

The findings in this report are subject to at least three limitations. First, NSSP and ESOOS case definitions might underestimate or overestimate opioid overdoses based on coding differences in hospitals, the availability of ICD-10-CM diagnostic codes, and the quality of chief complaint data (*13*). Consequently, analyses focused on comparison of trends by region and state, not of absolute rates. Findings should be verified against other data sources, and trends are expected to change slightly as visit data are updated. Second, hospital participation in NSSP varied across quarters; therefore, results could be related to changes in hospital participation. Finally, findings are not generalizable to areas not participating in NSSP or ESOOS.

With the rapidly evolving opioid overdose epidemic, ED data can serve as an early warning system, alerting communities to changes in prevalence of overdoses and permitting a timely, informed, and localized response that could facilitate a more rapid and coordinated response including targeting of resources (e.g., increase naloxone supply to affected areas), and issuance of emergency health alerts or advisories. EDs also can serve as a point of intervention for persons who experience an overdose and are at higher risk for a subsequent overdose. Educating ED physicians and staff members about appropriate services for immediate care and treatment and post-overdose protocols are important to preventing future overdoses among their patients. ED physicians could assess history of prescription drug use during care by accessing data from prescription drug monitoring programs and provide education to patients. Post-overdose protocols can help prevent subsequent overdose by providing naloxone and connecting patients with case management services or peer navigators to help link them into treatment and harm reduction services, including syringe services programs (*12*). Opioid overdoses continue to increase in most jurisdictions, and rapid response efforts and a multisectoral approach are needed to reduce and prevent overdoses and their associated morbidity and mortality.

**Key Points**

• During July 2016–September 2017, emergency department (ED) visits among those aged ≥11 years for opioid overdoses in the United States increased 29.7% overall and 34.5% in 16 states with high prevalence of overdose mortality. Significant rate increases were found in five Midwest region states (largest in Wisconsin [109%]) and in three Northeast region states (largest in Delaware [105%]); nonsignificant decreases (<10%) were found in three Northeast states. In the Southeast, rates increased in North Carolina (31%) and decreased in Kentucky (15.0%).

• Every demographic group reported substantial rate increases, including males (30%) and females (24%) and persons in all age groups (25–34 [31%]; 35–54 [36%], and ≥55 [32%] years).

• The highest opioid overdose rate increases occurred in large central metropolitan areas (a population of ≥1 million and covering a principal city).

• ED syndromic data can serve as an early warning system to alert communities of changes in opioid overdoses because of the rapid availability of this data (i.e., can be viewed within 24–48 hours of an ED visit).

• Treatment in EDs for drug overdose provides opportunities for intervention and prevention, which require coordination among all involved health care providers, allied health professionals, and agencies.

• Additional information is available at https://www.cdc.gov/vitalsigns/.

## Footnotes

* Emergency department visits are determined by considering facilities that are categorized as “emergency” and for patients who are deemed “emergency” status and excludes patients designated as only inpatient or only outpatient.

† NSSP’s BioSense platform launched in 2003 to establish a national public health surveillance system for early detection and assessment of potential bioterrorism-related illness. It has expanded to track infectious diseases and injuries. https://www.cdc.gov/nssp/biosense/index.html.

§ Enhanced State Opioid Overdose Surveillance (ESOOS) (#CDC-RFA-CE16-1608) started in 2016 and now funds 32 states and the District of Columbia to increase the timeliness of all suspected nonfatal drug, opioid, and heroin opioid overdose reporting (e.g., emergency department); increase the timeliness and comprehensiveness of fatal opioid overdose reporting and associated risk factors; and disseminate findings to stakeholders working to prevent or respond to opioid-involved overdoses. https://www.cdc.gov/drugoverdose/foa/state-opioid-mm.html.

¶ Analyses were intended to include nonfatal opioid overdose visits with unintentional and undetermined intents. ED visits resulting in death were not excluded, but accounted for only 1% of total opioid overdose ED visits in ESSENCE during the study period (data not shown). Though not explicitly excluded, *International Classification of Diseases, Tenth Revision, Clinical Modification* diagnosis codes or chief complaint text fields that mention intentional or assault-related opioid overdoses were not included because trends in unintentional overdoses are expected to differ from intentional overdoses. In addition, intentional opioid overdose is not as common as unintentional overdose. During 2006–2011, 26.5% of opioid overdoses were intentional; whereas, 53.5% and 20.0% were for unintentional and undetermined intents, respectively.

** Listed are the states within regions that currently share data with NSSP and had data available for the timeframe in this study. The Northeast region includes HHS Regions 1 (Maine, Massachusetts, New Hampshire, Rhode Island, and Vermont), 2 (New Jersey and New York), and 3 (District of Columbia, Maryland, Pennsylvania, Virginia, and West Virginia); the Southeast region includes HHS Region 4 (Alabama, Florida, Georgia, Kentucky, Mississippi, North Carolina, South Carolina, and Tennessee); the Southwest region includes HHS Region 6 (Arkansas, Louisiana, New Mexico, and Texas); the Midwest region includes HHS Regions 5 (Indiana, Illinois, Michigan, Minnesota, Ohio, and Wisconsin) and 7 (Iowa, Kansas, Missouri, and Nebraska); and the West region includes HHS Regions 8 (Colorado, Montana, North Dakota, and Utah), 9 (Arizona, California, and Nevada) and 10 (Alaska, Idaho, Oregon, and Washington). Some of the states listed above do not provide data for the entire state; for example, Texas (Region 6) has data from 50 counties; Iowa (Region 7) has data from one county; Colorado (Region 8) has data from three counties; and California (Region 9) has data from seven counties.

†† A 3–4 week delay usually occurs in the submission of discharge diagnosis codes that might affect the ability of the state case definitions to detect overdoses when free text information is unavailable or sparse. In addition, availability and completeness of data vary across the approximately 2,500 EDs with chief complaint text and discharge diagnosis codes missing in 15% and 46% of ED visits in NSSP, respectively.

§§ Additional information on the development of the opioid overdose case definition is available upon request to the corresponding author.

¶¶ https://surveillance.cancer.gov/joinpoint/.

*** State and local health departments using NSSP have access to their own detailed data (i.e., case-level data) and aggregate national and regional data and can share their detailed data with any other jurisdiction or CDC. CDC has access to all of the detailed data for operations and management purposes. In addition, for surveillance purposes, CDC can run queries against a subset of data elements and generate and report regional and national results. With approval, CDC also conducts collaborative analyses with jurisdictions and can access their detailed data. With ESOOS, states could allow CDC access to their data in NSSP for analysis in ESSENCE, or they could provide data (either syndromic or hospital billing) in different formats.

††† The six classification levels for counties were 1) large central metro: part of a metropolitan statistical area with ≥1 million population and covers a principal city; 2) large fringe metro: part of a metropolitan statistical area with ≥1 million population but does not cover a principal city; 3) medium metro: part of a metropolitan statistical area with ≥250,000 but <1 million population; 4) small metro: part of a metropolitan statistical area with <250,000 population; 5) micropolitan (nonmetro): part of a micropolitan statistical area (has an urban cluster of ≥10,000 but <50,000 population); and 6) noncore (nonmetro): not part of a metropolitan or micropolitan statistical area.

§§§ Additional information is available on estimates of drug overdose deaths in 2017 compared with 2016 in Massachusetts (https://www.mass.gov/files/documents/2017/11/15/2017-annual-update-action-items-gov-working-group.pdf), New Hampshire (https://www.dhhs.nh.gov/dcbcs/bdas/documents/dmi-october-2017.pdf), Maine (http://www.maine.gov/ag/news/article.shtml?id=765461) and Rhode Island (http://www.health.ri.gov/data/drugoverdoses/) as well as Delaware, Maine, and Pennsylvania (https://www.cdc.gov/nchs/nvss/vsrr/drug-overdose-data.htm).

¶¶¶ Additional information is available on Rhode Island’s plan on addiction and overdose (http://www.health.ri.gov/news/temp/RhodeIslandsStrategicPlanOnAddictionAndOverdose.pdf), State of Rhode Island Executive Order 17-07 (http://www.governor.ri.gov/documents/orders/ExecOrder-17-07-07122017.pdf) and Commonwealth of Massachusetts governor’s working group on opioid overdose: action items (https://www.mass.gov/files/documents/2017/11/15/2017-annual-update-action-items-gov-working-group.pdf).
